# Health literacy development of Primary Health Care patients: qualitative research

**DOI:** 10.1590/0034-7167-2024-0154

**Published:** 2024-12-16

**Authors:** Bruna Midori Sonoda, Carla Baioni Bonadio, Caroline Krauser

**Affiliations:** I Hospital de Amor de Barretos. Barretos, São Paulo, Brasil; II Universidade Federal de São Paulo. São Paulo, São Paulo, Brasil

**Keywords:** Health Literacy, Chronic Disease, Primary Health Care, Qualitative Research, World Health Organization., Alfabetización en Salud, Enfermedad Crónica, Atención Primaria de Salud, Investigación Cualitativa, Organización Mundial de la Salud.

## Abstract

**Objectives:**

to identify the process of health literacy development among primary care patients, relating it to their self-care practices.

**Methods:**

qualitative, prospective research with 22 patients from two Family Health Strategy units. Data were obtained through individual semi-structured interviews, examined through descriptive statistics and thematic content analysis.

**Results:**

the results discuss how participants learn about health and how this resonates in their behaviors, culminating in two thematic categories: “Health knowledge construction”; and “Dialogue between health knowledge construction and patient care actions”.

**Final Considerations:**

health knowledge is developed mainly through interpersonal relationships, mediated by health professionals through bonding and communication. Community educational actions and training of health professionals in communication can promote health literacy and self-care among patients.

## RESUMEN

**Objetivos:**

identificar el proceso de desarrollo de la alfabetización en salud entre pacientes de atención primaria, relacionándolo con sus prácticas de autocuidado.

**Métodos:**

investigación cualitativa, prospectiva, con 22 pacientes de dos unidades de la Estrategia de Salud de la Familia. Los datos fueron obtenidos a través de entrevistas individuales semiestructuradas, examinadas mediante estadística descriptiva y análisis de contenido temático.

**Resultados:**

los resultados discuten cómo los participantes aprenden sobre salud y cómo esto resuena en sus comportamientos, culminando en dos categorías temáticas: “La construcción del conocimiento en salud”; y “El diálogo entre la construcción de conocimientos en salud y las acciones de atención al paciente”.

**Consideraciones Finales:**

el conocimiento en salud se desarrolla principalmente a través de relaciones interpersonales, mediadas por los profesionales de la salud a través del vínculo y la comunicación. Acciones educativas comunitarias y formación de profesionales de la salud en comunicación pueden promover la alfabetización en salud y el autocuidado en los pacientes.

## INTRODUCTION

Health literacy proposes empowering patients over their health condition, enabling shared decision-making with the health team. Gaps and difficulties in health literacy processes have been associated with an increase in unfavorable clinical outcomes^(1)^. In this direction, the World Health Organization (WHO) has developed four manuals on the subject. In the first volume, some dimensions are highlighted as important in health literacy development, such as identifying how patients acquire knowledge about health, how they put this knowledge into practice, how political and social contexts influence this development, among others. With this, the WHO warns of the need to know how people acquire health knowledge before developing strategies for health promotion and prevention^(1,2)^.

At the moment, studies on the subject have been carried out predominantly in developed countries, and have prioritized quantitative analyses to measure health literacy based on the development of scales, such as the Test of Functional Health Literacy In Adults (TOFHLA) and Rapid Estimate of Adult Literacy in Medicine (REALM)^(3)^. In the Brazilian context, validity studies of scales such as the Short Assessment of Health Literacy for Portuguese-speaking Adults (SAHLPA)^(4)^ and the Short Test of Functional Health Literacy In Adults (STOFHLA) also predominate^(5)^.

In 2018, a Brazilian study investigated the relationship between health literacy and sociodemographic factors, self-perception of health and quality of life in Primary Health Care (PHC) users, finding a significant association between low education and inadequate health literacy^(6)^. In qualitative research in PHC in Brazil, a study on health literacy among older adults that showed satisfaction with the information received in PHC units^(7)^ and another that assessed users’ perceptions of health promotion and prevention activities carried out by students in these units stand out^(8)^.

However, since health literacy is a concept that encompasses complex skills and highlighting the importance highlighted by the WHO of assessing how people acquire health knowledge, there is a gap in analyzing how health literacy is developed in the context of the Brazilian population, of which three out of ten Brazilians are considered functionally illiterate^(9)^. Added to this is the importance of understanding this phenomenon in PHC, which is the main place of care for patients with chronic non-communicable diseases. Thus, the current study aimed to identify the process of developing patients’ health literacy, relating it to their self-care practices.

## OBJECTIVES

To identify how patients develop health literacy and relate these findings to their self-care actions in the PHC context.

## METHODS

### Ethical aspects

The study complied with national and international ethical precepts for research involving human beings, and was approved by the *Hospital de Câncer de Barretos - Fundação Pio XII* Research Ethics Committee. All participants signed the Informed Consent Form (ICF) and were instructed regarding their anonymity and their freedom to withdraw their data from the research at any time. To preserve participant anonymity, they were identified with the letter P, followed by a number, referring to the order in which they were interviewed and, finally, the initial letter of the health unit belonging to patients.

### Theoretical framework

Analyses on health literacy used in this study were carried out from the perspective of health literacy manuals developed by the WHO^(2)^.

### Study design

This is a qualitative, prospective study that followed the COnsolidating criteria for REporting Qualitative research (COREQ) guidelines^(10)^.

### Methodological procedures

The interviews were collected by the main authors of the study who, during the collection period, were residents in family and community medicine and, according to the organization of the residency, spent two years working in Family Health Strategy (FHS) units. This longitudinal work allowed them to form significant bonds with the population covered by the units and to perceive common weaknesses and potentialities within their respective health units, culminating in a curiosity about the development of patients’ health literacy, which is so important for health care, especially in primary care.

Two instruments were used to collect data: a questionnaire with patient sociodemographic data; and a semi-structured interview script constructed by the researchers, with ten guiding questions. The interviews were recorded individually with each participant after their consent, using the researchers’ personal recorder. Afterwards, the audios were stored in REdCap^(11)^.

### Study setting

The study was conducted in two FHS units, one of which was made up of a FHS team, covering a population of approximately 4,500 inhabitants, and the other, made up of three health teams, covering 9,684 individuals.

### Data source

Study participants are users of two FHS units, who were included because they were from the area covered by the FHS units of the study, were between 18 and 70 years old and had at least one chronic disease. Patients with severe neurological or psychiatric conditions and patients under exclusive home care were excluded from the study.

Firstly, the researchers identified the three most frequent chronic diseases in each reference team of the FHS units in the study, which became the chronic diseases investigated in this study. Selection was carried out according to Flick’s (2009) recommendations for sample selection in qualitative research. Among the various possibilities for recruitment in qualitative research indicated by the author, the researchers used formal sampling, chosen due to limited time to complete the research^(12)^.

According to the author, in qualitative research, it is crucial to select participants based on relevant criteria to obtain homogeneous content, in order to demonstrate cohesion in the analysis of reports provided by users as well as highlight the diversity between the different attributes of research participants^(12)^. To achieve this, participants with extreme or deviant cases for each health condition were initially chosen, followed by typical cases. Within each chronic health condition, at least two participants were selected with distinct variables, such as age, biological sex, years of education and whether or not they had a companion. Patients who met the inclusion criteria were selected based on the researchers’ experience and knowledge of the population being monitored and through case discussions in team meetings, until the criteria were in accordance with the author’s recommendations for formal sampling^(12)^. As a result, 22 patients were recruited between August and September 2024.

### Data collection and organization

Firstly, a script was prepared with personal and sociodemographic data of each participant and then a semi-structured interview was conducted with the following guiding questions: what do you understand about your disease? How do you take care of your health? How did you learn the information about your disease? Can you understand all the information that health professionals give you during consultation? What difficulties do you have in taking care of your health? When the doctor writes a prescription, do you understand it? How do you feel about the decisions for your treatment? When you receive information through another means, other than the health team, do you talk to the team about it? How do you see access to our health unit? The interviews took place between 10/01/2023 and 12/01/2023, and were conducted in person at the FHS of reference for each participant, lasting 12 to 38 minutes. Each interview was recorded on the researchers’ own recorder and stored in REDcap, with the presence of the researcher and the participant.

### Data analysis

**Figure 1:**
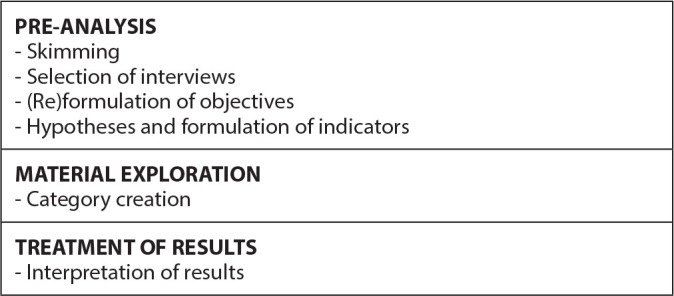
Stages of Bardin’s thematic content analysis

Participant sociodemographic characterization was presented through descriptive statistics generated by REDCap^(11)^, presented in graphs and tables. The interviews were analyzed based on the theory of thematic content analysis proposed by Bardin^(13)^ and interpreted in light of the manual on health literacy developed by the WHO^(2)^. This process began with pre-analysis, in which we sought, through skimming, to familiarize ourselves with the data, choosing and highlighting passages with similar meanings. In parallel, we resumed an in-depth reading of the manuals, noting the dialogue between the results obtained in the interviews and the dimensions presented in the first volume of the WHO manual on health literacy, which explains how users acquire health knowledge and how they put it into practice^(2)^. Hence, it was possible to identify two thematic categories, which were submitted to the authors’ interpretation. Figure 1 illustrates the stages contemplated in the analysis.

## RESULTS

### Participant characteristics

Table 1 shows research participant sociodemographic characteristics. Table 2 shows the average number of years lived with the underlying disease.

**Table 1:** Research participant characterization

Variable	Category	n	%
Sex	Female	12	54.5
	Male	10	45.5
Age	18 to 39 years	3	13.6
	40 to 59 years	11	50.0
	Over 60 years	8	36.4
Marital status	Married	9	40.9
	Single	7	31.8
	Common-law marriage	3	13.6
	Divorced	2	9.1
	Widowed	1	4.5
Health unit	Ibirapuera	11	50.0
	Derby	11	50.0
Education	Incomplete elementary school	6	27.3
	Complete elementary school	3	13.6
	High school	11	50.0
	Higher education	2	9.1
Occupation	Retired	9	40.9
	Self-employed	4	18.2
	CLT	3	13.6
	Unemployed	1	4.5
	Housewife	5	22.7
Monthly family income	Less than 1 minimum wage	2	9.1
	From 1 to 3 minimum wages	14	63.6
	From 3 to 5 minimum wages	3	13.6
	More than 5 minimum wages	3	13.6
Underlying disease	Hypertension	13	59.1
	Diabetes mellitus	9	40.9
	Major depressive disorder	9	40.9
	Others	11	50.0
Health services attended	Health center	22	100.0
	Outpatient clinic	9	40.9
	Private doctor	8	36.4
	Other health care	4	18.2
Frequency in health services	Once a year	2	9.1
	2 to 5 times a year	10	45.5
	Every 2 months	1	4.5
	Once a month	4	18.2
	Every week	5	22.7
Report of difficulty in access	No	20	90.9
	Yes	2	9.1
Receives assistance from family members	No	8	36.4
or caregivers	Yes	14	63.6

*CLT - Consolidation of Labor Laws.*

**Table 2:** Mean years lived with the underlying disease

Underlying disease	Mean years lived
Diabetes mellitus	10.5 years
Hypertension Major depressive disorder	16.3 years 11.2 years

### Thematic categories of interviews

Two categories emerged from the interviews conducted: 1) Health knowledge construction; and 2) Dialogue between health knowledge construction and patient care actions.

### Health knowledge construction

According to the reports of research participants, knowledge construction for health literacy is established in four ways: through interpersonal relationships; through social media; through bodily sensations; and through relationships with health professionals. Among them, the most evident way of constructing health literacy was through personal experiences with family, friends or acquaintances and the beliefs that emerge from these relationships:

*I was always dealing with it, because I took care of my elderly mother, for many years, right? I took care of everything for ten years* [...]. (P3D)

*I had a lot of experience, because my husband had heart surgery twice* [...] *and that rush, always with him too, and with my mother, taking care of everything* [...] *so we deal with a lot of health problems, right?* (P3D)

*Because at work there are a lot of heart problems. I talked to everyone* [...] *but why did you show up with your problem? So, we talk, even about medicine, which doctor are you going to? So, you’ll gain a lot of knowledge.* (P5D)

*My colleagues, I know a lot of people with diabetes, they talk so much, then things start to add up, check mate, you know? The eyesight, the tired legs, the tired body.* (P8D)

Even when accompanied by guidance from a health professional, knowledge construction for health literacy is facilitated when the professional is a family member or someone they know:

*Friends from work, from the Basic Health Unit and from the family unit also have family members who are doctors.* (P7D)

*My niece is a secretary. She is now a nurse. She works at the reception desk at Santa Casa. My sister is now a social worker. She also teaches me a lot. I have doubts, but at home I have an introduction, is that what I say? An interpreter, who is Joana. She has already studied nursing, worked in the health area* [...]. (P4D)

*My daughter is a nursing technician, today she works in the laboratory, but she worked more in the administrative area. So, I ask her to ask the doctor everything, there is always guidance.* (P3D)

Sometimes, this construction, through family relationships, generates doubts and can generate a certain disbelief in relation to guidelines on chronic diseases:

*My mother died at 94 years old, eating only pork fat. The only thing that happened was that she lost her sight, she couldn’t see anymore. She was lucid, she didn’t have diabetes. My sister had it, my brother had it and my sister’s daughters already have it. So, I don’t know, is it sedentary or what else can it be.* (P3D)

*I don’t even know what to say about my illness, because my mother never drank, never smoked, never did anything, she has diabetes and even high blood pressure* [...] *so, I don’t know.* (P9D)

The internet also appears as an ally in knowledge construction for participants’ health literacy:

*Because I also do a lot of research on Google* [...] *on Google, because you have to research. Don’t be so ignorant about health, your own health, right?* (P4D)

*On Google or YouTube. There are always reports of people explaining it. But even though the disease is the same, it’s just that each person is different.* (P8I)

*On the internet, I see the lesions that diabetes causes and I tell the doctor. I was a little scared.* (P11I)

However, patients often compare information on the internet with the advice of health professionals:

*I only feel trustworthy when I see a health center or some health unit, because I even look for information like that on the internet. The other day it came up that diabetes is caused by a worm in the pancreas, and then you’re like, “Oh my God”.* (P1I)

*I look for it because I don’t like to do things on my own, I try to find out, because there are things on the internet that you can’t* [...]. (P4I)

Guidance and connections with health professionals also appear to be important in building knowledge for participants’ health literacy:

*Without the help of a doctor, without the help of a professional in this area, you can’t do anything, you’ll just read, but you won’t do anything, you need to have someone to help you, to show you the right path.* (P3I)

*I go to the pharmacist and always mark exactly how to take it, stick a little piece of paper, time, how to take it, whether it’s before a meal, whether it’s after.* (P3D)

*Look, doctor, I’m not one to research things on the internet, no. I have the information I have, it’s from my doctors who follow me, right?* (P4I)

*I’ve gotten so used to the doctor that I try to come on the day she’s there to solve it. I feel like: if I have a problem, I can come here tomorrow morning and it will be solved. I’m very happy.* (P6D)

Finally, another way to build health literacy is through the perception of one’s own body:

*My head gets cloudy sometimes and my head feels bad, then I know it’s a little high.* (P2D)

*Sometimes, a little dizziness, nausea, shortness of breath. Sometimes, tachycardia* [...] *until I discovered that it was anxiety.* (P5I)

*Because when it goes up uncontrollably, it makes you feel bad, it makes you nauseous. And when it goes down, it makes me desperately hungry, I even eat things I don’t like. And I feel very weak, sometimes it even feels like I’m going to faint from dizziness.* (P8I)

### Dialogue between health knowledge construction and patient care actions

When health literacy construction occurs mainly through interpersonal relationships, health care is linked to these relationships:

*Because my neighbor, she was like that. I’m not feeling anything, I’m not going to take it. I was like that too* [...] *but then, she lay down, had a facial hemorrhage. So, from now on, oh, I’m not without my medicine under any circumstances.* (P6D)

*I want them to be proud of me, because they say they’re there thinking about me here.* [...] *my son said, “Hey, come here so we can buy the medicine for you”* [...] *I took it and said, “Well, I’m not going to disappoint him, right? I’m going to do it right”.* (P6D)

[...] *she found out she had cancer, right? It was too late, you know? So, even because I’ve been with her through all this, I try to take good care of my health, like, as much as I can, you know?* (P6I)

*Because my neighbor, she went to do it because I didn’t have anyone to go with me, right? So, my neighbor is doing it because she’s pre-diabetic and needs to do it and she’s taking me. Because there I am alone, I am afraid, I will not go* [...]. (P2D)

*Then, I was talking to a friend of mine, and she said, “No, Regina, there’s no danger, they won’t let you drown.” It gave me confidence* [...] *she said, “You can go with me, we’ll come together”* [...]. (P2D)

*My daughter is studying medicine, she’s always on our case, she keeps a close eye on us, you know? She sees how we’re doing, she sees all the tests, you know? She sees what we’re eating, what we’re not eating, she keeps an eye on us. She keeps a close eye on us too.* (P6I)

*I hear at the bar, you know? That there are a lot of people who have diabetes there, that’s why I don’t understand the diabetes thing. I can’t eat sweets, I can’t eat anything. There are others who go there, who drink pinga* [Brazilian beverage] *with coke and are diabetic too. Why is one different from the other?* (P6D)

Although social media appears to be a contributor to health knowledge construction, it does not appear to be an aid to the care process. However, the relationship between knowledge acquired by health professionals appears to be an important contributor, and is permeated by factors such as bonding, listening, seeing professionals as an authority figure, and professionals’ body and verbal language:

*She only asked for the tests. She said, “You have to do this thing”. I get really down, worried* [...] *it makes me afraid to do the test and find out* [...] *so I say, “I’m not going to do this test”* [...]. (P4D)

[...] *a decision I made together was made by me and my psychologist, we were discussing the next steps, and I realized that the place I was living was not good. I went there and moved, I changed the activities I was doing during the day. Over time, I started waking up earlier again, going for walks, exercising.* (P1I)

[...] *and I was working normally, and they called me to do a regular check-up* [...] *the doctor took my blood pressure, listened to my heartbeat, and then told me I had to wait, because my blood pressure had changed* [...] *another person quickly attended to me and called an ambulance, and said I had to go straight to the emergency room, and I thought I was dying, I froze* [...]. (P5D)

*I feel fine, he’s a specialist, he knows, he’s studied, right? So, he advised me to change, I have to change, right?* (P3D)

[...] *because he’s there making himself available and seeing part of the process I’m going through. So, I believe he’s prescribing it to me because it’s going to do me good, right?* [...]. (P11D)

In this regard, the professional-patient relationship often initiates the motivation for health care, and the sensations perceived in the body, as well as interpersonal relationships, function as facilitators or hinders in maintaining this care:

*Now, I’m participating in the group, right? A women’s group. It’s something that’s really helping me learn techniques, right? Meditation, breathing, we talk. So, it’s doing me good. I’m also having individual consultations with her.* (P11D)

*Oh, I try to spend time with my family on the weekends, to pay more attention to my children* [...] *because I realize that the emotional part, anxiety, is a factor that changes a lot* [...] *at least on the weekends, I try to do some things differently* [...] *go for a walk, knowing what we have to do to feel better.* (P7D)

*Yes, I’m going for walks and my psychiatrist even advised me to go to the gym, because I was very quiet. But otherwise, I’m going to therapy. I can’t do without it. We have groups, a wonderful support group, so I don’t stop going. If I stopped going, I wouldn’t be here anymore* [...]. (P4I)

*Because if there is a treatment, a care for this, why not do it? Walk a little, eat more fruit* [...] *and be calm. Try to stay calm too, because that contributes a lot to these feelings.* (P7I)

*I stopped on my own, when I see that I’m getting really bad, I cry, and then it goes away a little* [...] *and the medicine improves one thing, but hinders the other. As for dating, I had no desire at all. So, I think the medicine helped at that time when I was crying too much, all the time, but I think that now there’s no need for it* [...]. (P10I)

Unlike social media, which appear as contributors to health knowledge construction, but not to care, faith and religion appear as factors that help in health care:

*I can tell you that every day we have to seek our faith* [...] *because without faith we don’t get anywhere* [...] *we read the Bible, we read the word, looking for a place to go. I think that’s the fundamental point. Not only for this, but for all the issues in our lives.* (P7I)

*Thank God, I got rid of all that, because I sought it in my faith* [...] *of course there is psychological and psychiatric help from doctors who are qualified for that. But fundamentally it’s this search, it’s a set of things that you have to do.* (P7I)

## DISCUSSION

In this study, we can see the alignment with the WHO manual regarding the dimensions of how health service users acquire knowledge and apply it in self-care practices. We realize that knowledge acquisition occurs predominantly through closer social interactions, including friends and family, especially if the latter are health professionals or if this learning emerges from the experience of individuals as caregivers of a family member with a health condition similar to their own. Dialogue allows individuals to share their experiences, concerns, seek emotional support and learn from each other’s experiences, demonstrating, according to WHO manuals, that health literacy development is a social practice^(2)^.

Knowledge construction for health literacy through interpersonal relationships motivated most of the interviewees to self-care. When they observed complications from chronic diseases in people close to them, they started to take better care of their diet or practice physical activity. However, for some, this relationship generated skepticism regarding the information provided by health professionals, comparing it with the reality experienced by their family members. One participant in this study mentioned a family member with unhealthy eating habits who had a long life without developing diabetes, questioning the relationship between healthy habits and chronic diseases.

This result is in line with studies aimed at understanding individuals’ health literacy^(1,2,14)^. The concept of health literacy mediators describes people who make their health literacy available to others, formally or informally, and highlights social support as one of the most important mediators of health literacy^(14)^, highlighting what the WHO manual on health literacy warns: the fact that health literacy is not an individual task and that friends and family communicate with health professionals on behalf of and in collaboration with the patients they assist^(2,14)^. The proximity of interpersonal relationships was also in line with those found in this study, with the nuclear family being the main mediator in health literacy construction, followed by friends and, finally, coworkers and members of support groups^(1)^.

These findings highlight the importance of initiatives such as the Patientand Family-Centered Care Model (PFCCM)^(15)^. However, recent studies show the difficulty of health professionals in including patients and family members in care decisions. In Brazil, research indicates the difficulty of integrating PFCCM into patient care^(15,16)^, in addition to having an insufficient number of studies in the area. Another important point in the relationship between health knowledge construction and interpersonal relationships is the importance of strategies within the patient community. Patients who participated in health education groups learned more about their conditions than those who did not participate^(1,14,17,18)^. Interventions that promote group support are also emphasized by the WHO health literacy manual^(2)^.

This study, like others on health literacy, showed that digital technology and social networks are fundamental tools in the dissemination of public health information^(1,14,19,20)^. Some patients interviewed consider the media to be one of the main sources of information acquisition, due to the speed and accessibility of content. However, there is a duality, since the population of this study sought to compare digital information with that provided by trusted health professionals, perceiving it as confusing, contradictory or even false.

Therefore, the results of this study support previous research, by identifying social media as an important factor in health literacy development. However, in this study, the media are viewed with suspicion and appear as secondary to interpersonal relationships. However, as the use of social media appears in participants’ statements, the importance of using it as an ally in health care is evident. National and international studies show that social media can provide reliable information, form support communities among users with the same health conditions, facilitate behavioral changes necessary to manage chronic conditions, train health professionals, and monitor and track patients’ habits^(1,19,20)^.

In line with studies on health literacy, participants in this study highlight the role of health professionals as facilitators of the learning process, with a good doctor-patient relationship being crucial for individuals to trust the information received. The quality of the health professional-patient relationship is related to trust, empathy, communication, listening and information sharing. However, studies show that not all professionals support health literacy development or encourage patients to interact with information before choosing treatment. Some create barriers that prevent the development of self-care skills^(1,14)^.

In this study, patients highlighted that observing health professionals’ body and verbal language is essential for decision-making, generating trust or distrust. Patients with less trust and connection with health professionals have higher levels of glycated hemoglobin and more cardiovascular events^(21,22)^. The main barriers to health literacy are poor communication skills among health professionals, which makes patients feel that they have not received enough information and have not been listened to. Some patients report that their information was not taken into account by professionals during consultation. By not taking into account the needs of patients themselves, behavior change becomes more difficult.

This fact appears in the reports of participants in this study. It is clear that participants are more likely to change their behavior when they realize that the information provided by health professionals is constructed jointly, based on the bond and their own needs. In contrast, when professionals are seen only as an authority figure, reports of behavior change do not accompany the discourse. In this regard, communication strategies, such as motivational interviewing (MI), are important allies in promoting these changes. In Brazil, MI is applied, in short, in the context of the use of psychoactive substances, and is still little used in the context of PHC^(23)^.

Another way of constructing knowledge for health literacy evidenced in this study was their own body perceptions, which were correlated to a given health condition. It is up to health professionals to encourage patients to develop self-awareness and guide them in interpreting such signs and symptoms so that they can differentiate between common symptoms and serious symptoms. The WHO states that activities that encourage moments of self-awareness favor health literacy construction and self-care promotion, such as Mindfulness and Yoga, which are considered important tools in self-care construction^(2,17,18)^.

Finally, faith, based mainly on the Christian religion, appears as a mediator of motivation for self-care in health, a fact not found in other qualitative studies on health literacy. It is known that religious practices and groups have a great influence on the community structure and, at times, participate in the promotion of preventive programs, such as rehabilitation centers, physical activity practices and health education^(24)^. Hence, religious places can be designed to distribute educational pamphlets, disseminate health information and even serve as a stage for health professionals to give educational lectures.

The current study highlights the need for trained professionals to explore and utilize patients’ prior knowledge about their chronic health conditions, especially their personal and interpersonal experiences. It is crucial to implement communication actions that reach communities and disseminate health information broadly, beyond individual consultations. Continuing education in communication techniques and motivation for behavior change can be an effective strategy. Complementary therapies, especially mind-body practices, are important allies, as they promote body self-knowledge and can be applied in groups.

### Study limitations

This study identified some difficulties. Firstly, conducting the interviews was hampered by the limitation of physical space, as one of the health units had inadequate infrastructure and a small room, without adequate ventilation, for conducting the interviews. Finally, the deadline for submitting the Residency Conclusion Work, combined with the practical workload required by the residency program, prevented the exploration of other aspects of health literacy, such as comparing health literacy construction indicated by participants with the opinions of health professionals who assist this population, in order to investigate discrepancies and similarities in the discourses as well as to carry out a content analysis before and after health education interventions in the same participants. These notes may constitute future fields of research needed in Brazil.

### Contributions to nursing, health or public policy

This study was the first to qualitatively investigate knowledge acquisition for health literacy in PHC patients in Brazil. Studies with similar objectives have been conducted outside Brazil, analyzing populations with different cultural habits, ethnicities, economies, geographies and social practices, without considering Brazilian specificities. This may explain why this study was the only one to report the contribution of religion to health literacy, highlighting the need for more Brazilian research. Although quantitative studies on the topic are important, qualitative research is crucial to capture the subjectivities of discourses, essential to understanding complex social phenomena such as health literacy.

## FINAL CONSIDERATIONS

The results indicate that the analysis of how health literacy is constructed in chronic non-communicable disease management is a continuous process of self-knowledge and self-management of one’s own health condition. Family members, friends and co-workers are also the main mediating agents in health literacy development and practice, sharing knowledge, experiences, skills and forming a support network in decision-making. Therefore, understanding family dynamics also allows us to understand the health literacy construct. The health professional-patient relationship is an important pillar for the information received by patients to be transformed into motivation for self-care. Thus, PHC, based on longitudinality and the person-centered approach (PFCCM), contributes so that the health professional-patient relationship is not a barrier, but rather a dyad alliance for health literacy construction.
