# Supplementary material for: Health literacy development of Primary Health Care patients: qualitative research
Source: Rev Bras Enferm. 2024 Dec 16;77(6):e20240154. doi: 10.1590/0034-7167-2024-0154 (PMC11654553; doi:10.1590/0034-7167-2024-0154)
Supplement: Translation

# Translation: Construção do letramento em saúde de pacientes da Atenção Primária à Saúde: uma pesquisa qualitativa

**DOI:** 10.1590/0034-7167-2024-0154pt

**Authors:** Bruna Midori Sonoda, Carla Baioni Bonadio, Caroline Krauser

**Affiliations:** I Hospital de Amor de Barretos. Barretos, São Paulo, Brasil; II Universidade Federal de São Paulo. São Paulo, São Paulo, Brasil

**Keywords:** Letramento em Saúde, Doença Crônica, Atenção Primária à, Saúde, Pesquisa Qualitativa, Organização Mundial da Saúde.

## Abstract

**Objetivos:**

identificar o processo do desenvolvimento do letramento em saúde entre pacientes da atenção primária, relacionando-o com suas práticas de autocuidado.

**Métodos:**

pesquisa qualitativa, prospectiva, com 22 pacientes de duas unidades de Estratégia Saúde da Família. Os dados foram obtidos através de entrevistas semiestruturadas individuais, examinadas através de estatística descritiva e análise temática de conteúdo.

**Resultados:**

os resultados discorrem sobre como os participantes aprendem sobre saúde e como isso ressoa em seus comportamentos, culminando em duas categorias temáticas: “A construção do conhecimento em saúde”; e “O diálogo entre a construção do conhecimento em saúde e as ações de cuidado dos pacientes”.

**Considerações Finais:**

o conhecimento em saúde é desenvolvido, principalmente, através das relações interpessoais, mediadas por profissionais de saúde através do vínculo e da comunicação. Ações educativas comunitárias e a capacitação de profissionais de saúde em comunicação podem promover o letramento em saúde e o autocuidado dos pacientes.

## INTRODUÇÃO

O letramento em saúde propõe o empoderamento do paciente sobre sua condição de saúde, possibilitando a tomada de decisão compartilhada com a equipe de saúde. Lacunas e dificuldades nos processos de letramento em saúde têm sido associadas ao aumento de desfechos clínicos desfavoráveis^(1)^. Nessa direção, a Organização Mundial da Saúde (OMS) desenvolveu quatro manuais sobre o tema. No primeiro volume, algumas dimensões são evidenciadas como importantes no desenvolvimento do letramento em saúde, como identificar como os pacientes adquirem o conhecimento sobre saúde, como colocam esse conhecimento em prática, como os contextos políticos e sociais influenciam esse desenvolvimento, entre outros. Com isso, a OMS alerta para a necessidade de conhecer como as pessoas adquirem o conhecimento em saúde antes de desenvolver estratégias para a promoção e prevenção em saúde^(1,2)^.

No momento, os estudos sobre a temática foram realizados predominantemente em países desenvolvidos, e priorizaram as análises quantitativas para mensurar o letramento em saúde a partir da elaboração de escalas, como *Test of Functional Health Literacy In Adults* (TOFHLA) e *Rapid Estimate of Adult Literacy in Medicine* (REALM)^(3)^. No contexto brasileiro, também predominam estudos de validação de escalas como *Short Assessment of Health Literacy for Portuguese-speaking Adults* (SAHLPA)^(4)^ e *Short Test of Functional Health Literacy In Adults* (STOFHLA)^(5)^.

Em 2018, estudo brasileiro investigou a relação entre letramento em saúde e fatores sociodemográficos, autopercepção de saúde e qualidade de vida em usuários da Atenção Primária à Saúde (APS), encontrando uma associação significativa entre baixa escolaridade e letramento em saúde inadequado^(6)^. Em pesquisas qualitativas na APS do Brasil, destacam-se estudo sobre letramento em saúde em idosos que evidenciou satisfação com as informações recebidas nas unidades de APS^(7)^ e outro que avaliou as percepções dos usuários sobre atividades de promoção e prevenção de saúde realizadas por estudantes nessas unidades^(8)^.

Contudo, sendo o letramento em saúde um conceito que engloba competências complexas e salientando a importância evidenciada pela OMS de se avaliar como as pessoas adquirem o conhecimento em saúde, há uma lacuna em analisar como o letramento em saúde é desenvolvido no contexto da população brasileira, da qual três a cada dez brasileiros são considerados analfabetos funcionais^(9)^. Soma-se a isso a importância de compreender esse fenômeno na APS, que consiste no principal local de atendimento para pacientes com doenças crônicas não transmissíveis. Nessa direção, o estudo atual tem como objetivo identificar o processo do desenvolvimento do letramento em saúde dos pacientes, relacionando-os com suas práticas de autocuidado.

## OBJETIVOS

Identificar como os pacientes desenvolvem o letramento em saúde e relacionar esses achados com suas ações de autocuidado no contexto da APS.

## MÉTODOS

### Aspectos éticos

O estudo respeitou os preceitos éticos nacionais e internacionais em pesquisa com seres humanos, sendo aprovado pelo Comitê de Ética em Pesquisa do Hospital de Câncer de Barretos - Fundação Pio XII. Todas as pessoas que participaram do estudo assinaram o Termo de Consentimento Livre e Esclarecido (TCLE), e foram orientadas em relação ao seu anonimato e à sua liberdade de retirar seus dados da pesquisa a qualquer momento. Para a preservação do anonimato dos participantes, eles foram identificados com a letra P, seguido de um número, referente à ordem em que ele foi entrevistado e, por fim, da letra inicial da unidade de saúde pertencente ao paciente.

### Referencial teórico

As análises sobre o letramento em saúde utilizadas neste estudo foram realizadas sob a ótica dos manuais de letramento em saúde elaborados pela OMS^(2)^.

### Tipo do estudo

Estudo de abordagem qualitativa, de carácter prospectivo, que seguiu as diretrizes do *COnsolidating criteria for REporting Qualitative research* (COREQ)^(10)^.

### Procedimentos metodológicos

As entrevistas foram coletadas pelas autoras principais do estudo que, no período de coleta, eram residentes de medicina de família e comunidade e, conforme a organização da residência, passaram dois anos atuando nas unidades de Estratégia Saúde da Família (ESF). Essa atuação longitudinal permitiu que elas formassem vínculos significativos com a população de abrangência das unidades e percebessem fragilidades e potencialidades em comum dentro de suas respectivas unidades de saúde, culminando em uma curiosidade sobre o desenvolvimento do letramento em saúde dos pacientes, tão importante para o cuidado em saúde, especialmente na atenção primária.

Para a coleta dos dados, foram utilizados dois instrumentos: um questionário com dados sociodemográficos dos pacientes; e um roteiro de entrevista semiestruturada construído pelas pesquisadoras, com dez questões norteadoras. As entrevistas foram gravadas individualmente com cada participante após seu consentimento, através do gravador pessoal das pesquisadoras. Após, os áudios foram armazenados no *software* REdCap^(11)^.

### Cenário do estudo

O estudo foi conduzido em duas unidades de ESF, sendo uma delas composta por uma equipe de ESF, que abrange uma população aproximada de 4.500 habitantes, e a outra, composta por três equipes de saúde, que abrange 9.684 indivíduos.

### Fonte de dados

Os participantes do estudo são usuários de duas unidades de ESF, que foram incluídos por ser da área de abrangência das unidades de ESF do estudo, ter entre 18 e 70 anos e ter ao menos uma doença crônica. Pacientes portadores de condição neurológica ou psiquiátrica grave e pacientes em acompanhamento domiciliar exclusivo foram excluídos do estudo.

Primeiramente, as pesquisadoras levantaram as três doenças crônicas mais frequentes em cada equipe de referência das unidades de ESF do estudo, que se tornaram as doenças crônicas investigadas neste estudo. A seleção ocorreu conforme as recomendações de Flick (2009) para seleção de amostragem em pesquisa qualitativa. Entre as diversas possibilidades de recrutamento em pesquisa qualitativa apontada pelo autor, as pesquisadoras utilizaram uma amostragem formal, escolhida em detrimento ao tempo limitado para a conclusão da pesquisa^(12)^.

Segundo o autor, na pesquisa qualitativa, é crucial selecionar os participantes com base em critérios relevantes para a obtenção de um conteúdo homogêneo, de forma a evidenciar coesão na análise dos relatos informados pelos usuários, bem como ressaltar a diversidade entre os diferentes atributos dos participantes da pesquisa^(12)^. Para alcançar isso, foram escolhidos, inicialmente, participantes com casos extremos ou desviantes para cada condição de saúde, seguidos dos casos típicos. Dentro de cada condição crônica de saúde, foram selecionados pelo menos dois participantes com variáveis distintas, como idade, sexo biológico, anos de escolaridade e ter ou não a presença de acompanhante. Os pacientes que atendiam aos critérios de inclusão foram sendo selecionados com base na experiência e no conhecimento das pesquisadoras sobre a população em acompanhamento e por meio de discussões de casos em reunião de equipe, até que os critérios estivessem de acordo com as recomendações do autor para uma amostragem formal^(12)^. Como resultado, 22 pacientes foram recrutados entre agosto e setembro de 2024.

### Coleta e organização dos dados

Primeiramente, foi elaborado um roteiro com dados pessoais e sociodemográficos de cada participante e, a seguir, foi realizada uma entrevista semiestruturada com as seguintes perguntas norteadoras: o que você entende sobre sua doença? Como você cuida da sua saúde? Como você aprendeu as informações sobre a sua doença? Você consegue entender todas as informações que os profissionais de saúde passam para você durante a consulta? Quais dificuldades você tem para cuidar da sua saúde? Quando o médico passa uma receita, você consegue entender? Como você se sente em relação às decisões para o seu tratamento? Quando você recebe uma informação por outro meio, sem ser pela equipe de saúde, você conversa com a equipe sobre isso? Como você vê o acesso à nossa unidade de saúde? As entrevistas ocorreram entre 01/10/2023 e 01/12/2023, e foram realizadas de forma presencial na ESF de referência para cada participante, tendo uma duração de 12 a 38 minutos. Cada entrevista foi gravada em gravador próprio das pesquisadoras e armazenada no *software* REDcap, contando com a presença da pesquisadora e do participante.

### Análise dos dados

A caracterização sociodemográfica dos participantes foi apresentada através da estatística descritiva gerada pelo *software* REDCap^(11)^, apresentada em gráficos e tabelas. As entrevistas foram analisadas a partir da teoria da análise temática de conteúdo proposta por Bardin^(13)^ e interpretadas à luz do manual sobre letramento em saúde desenvolvido pela OMS^(2)^. Esse processo se deu início com a pré-análise, na qual buscamos, através da leitura flutuante, nos familiarizarmos com os dados, escolher e grifar os trechos com sentidos semelhantes. Em paralelo, retomamos a leitura aprofundada dos manuais, notando o diálogo dos resultados obtidos nas entrevistas com as dimensões apresentadas no primeiro volume do manual da OMS sobre letramento em saúde, que explicita sobre como os usuários adquirem conhecimento em saúde e como os colocam em prática^(2)^. Com isso, foi possível identificar duas categorias temáticas, que foram submetidas à interpretação dos autores. A Figura 1 ilustra as etapas contempladas na análise.

**Figura 1:** Etapas da análise temática de conteúdo de Bardin

## RESULTADOS

### Características dos participantes

A Tabela 1 mostra a caracterização sociodemográfica dos participantes da pesquisa. A Tabela 2 mostra a média de anos vividos com a doença de base.

**Tabela 1:** Caracterização dos participantes da pesquisa

Variável	Categoria	n	%
Sexo	Feminino	12	54,5
	Masculino	10	45,5
Idade	18 a 39 anos	3	13,6
	40 a 59 anos	11	50,0
	Acima de 60 anos	8	36,4
Estado civil	Casado	9	40,9
	Solteiro	7	31,8
	União estável	3	13,6
	Divorciado	2	9,1
	Viúvo	1	4,5
Unidade de saúde	Ibirapuera	11	50,0
	Derby	11	50,0
Escolaridade	Ensino fundamental incompleto	6	27,3
	Ensino fundamental completo	3	13,6
	Ensino médio	11	50,0
	Ensino superior	2	9,1
Ocupação	Aposentada	9	40,9
	Autônomo	4	18,2
	CLT	3	13,6
	Desempregada	1	4,5
	Do lar	5	22,7
Renda familiar mensal	Menos de 1 salário mínimo	2	9,1
	De 1 a 3 salários mínimos	14	63,6
	De 3 a 5 salários mínimos	3	13,6
	Mais de 5 salários mínimos	3	13,6
Doença de base	Hipertensão arterial	13	59,1
	Diabetes mellitus	9	40,9
	Transtorno depressivo maior	9	40,9
	Outros	11	50,0
Serviços de saúde frequentados	Posto de saúde	22	100,0
	Ambulatório	9	40,9
	Médico particular	8	36,4
	Outro atendimento de saúde	4	18,2
Frequência no serviço de saúde	1 vez ao ano	2	9,1
	2 a 5 vezes no ano	10	45,5
	A cada 2 meses	1	4,5
	1 vez por mês	4	18,2
	Toda semana	5	22,7
Relato de dificuldade de acesso	Não	20	90,9
	Sim	2	9,1
Recebe auxílio de familiares	Não	8	36,4
ou cuidadores	Sim	14	63,6

*CLT - Consolidação das Leis do Trabalho.*

**Tabela 2:** Média de anos vividos com a doença de base

Doença de base	Média de anos vividos
Diabetes mellitus	10,5 anos
Hipertensão arterial sistêmica Transtorno depressivo maior	16,3 anos 11,2 anos

### Categorias temáticas das entrevistas

Duas categorias emergiram das entrevistas realizadas: 1) Construção do conhecimento em saúde e 2) O diálogo entre a construção do conhecimento em saúde e as ações de cuidado dos pacientes;

### A construção do conhecimento em saúde

Dentre o relato dos participantes da pesquisa, a construção do conhecimento para o letramento em saúde se institui a partir de quatro formas: através das relações interpessoais, das mídias sociais, das sensações corporais e da relação com profissionais de saúde. Dentre elas, a forma mais evidente de construção do letramento em saúde foi através da vivência pessoal com familiares, amigos ou conhecidos e das crenças que emergem a partir dessas relações:

*Eu sempre ia lidando, porque eu cuidei da minha mãe de idade, muitos anos, né. 10 anos eu cuidava de tudo* [...]. (P3D)

*Eu tive muita experiência, porque o meu marido, ele operou do coração duas vezes* [...]*e aquela correria, sempre com ele também. E, com a minha mãe, cuidando de tudo* [...]. *Então a gente lida com muitos problemas de saúde, né?* (P3D)

*Porque no trabalho tem muito cardíaco. Conversava com todo mundo* [...] *mas por que você apareceu com o seu problema? Então a gente fica conversando. Até sobre remédio, qual que é o médico que você vai? Então você vai ter muito conhecimento.* (P5D)

*Os meus colegas, conheço bastante gente de diabetes, eles falam tanta coisa, aí as coisas vai batendo, check mate, entendeu, as vistas, a perna cansada, o corpo cansado.* (P8D)

Mesmo quando acompanhada da orientação de um profissional de saúde, a construção do conhecimento para o letramento em saúde é facilitada quando o profissional é um membro da família ou um conhecido da mesma:

*Os amigos do trabalho, da unidade básica e familiar também, têm familiar médico na família.* (P7D)

*Minha sobrinha é secretária. Ela é agora enfermeira. Ela é da recepção da Santa Casa. A minha irmã, ela é hoje assistente social. Ela me ensina também muita coisa. Eu tenho dúvida. Mas, ali em casa, eu tenho uma introdução, é o que eu falo? Um intérprete, que é a Joana. Ela já fez enfermagem, trabalhou na área de saúde* [...]. (P4D)

*Minha filha, ela é técnica de enfermagem, hoje ela tá no laboratório, mas trabalhou mais na parte administrativa. Então peço pra ela perguntar pro médico, tudo assim, tem sempre uma orientação.* (P3D)

Por vezes, essa construção, através das relações familiares, gera dúvidas e pode gerar certa descrença em relação às orientações acerca das doenças crônicas:

*A minha mãe morreu com 94 anos só comendo gordura de porco. A única coisa que fez foi que ela perdeu as vistas, não enxergava mais. Ela era lúcida, não tinha diabetes. Já minha irmã teve, meu irmão teve e as filhas da minha irmã já têm. Então, eu não sei, é sedentário ou como que pode ser.* (P3D)

*A minha doença, eu nem sei o que falar, porque minha mãe nunca bebeu, nunca fumou, nunca nada, tem diabetes e tem até pressão também* [...] *então, não sei.* (P9D)

A internet também aparece como aliada na construção do conhecimento para o letramento em saúde dos participantes:

*Porque eu pesquiso muito no Google também* [...] *no Google, porque você tem que pesquisar. Não ficar tão leiga na parte da saúde, sua própria saúde, né?* (P4D)

*No Google ou no YouTube. Sempre tem relatos de pessoas explicando. Mas, assim, que mesmo a doença se é igual, é que cada pessoa é de um jeito.* (P8I)

*Na internet, eu vejo as lesões que causa a diabetes e falo para a doutora. Eu fiquei meio assustado.* (P11I)

No entanto, os pacientes costumam confrontar as informações da internet com as orientações de profissionais de saúde:

*Eu só sinto confiável quando eu vejo um postinho ou alguma unidade de saúde, porque eu até busco informação assim na internet. Outro dia apareceu que diabetes é causada por um verme no pâncreas, aí você fica assim: “Meu Deus do céu”.* (P1I)

*Procuro porque eu não gosto de fazer coisa por minha conta, eu procuro saber, porque tem coisas da internet que não pode* [...]. (P4I)

A orientação e o vínculo com profissionais de saúde também aparecem como importantes na construção do conhecimento para o letramento em saúde dos participantes:

*Sem a ajuda de um médico, sem a ajuda de um profissional nessa área, você não faz nada, simplesmente vai ler, mas não vai fazer nada, você precisa ter alguém que auxilie, que dê um caminho certo.* (P3I)

*Eu vou no farmacêutico e sempre marco certinho o jeito de tomar, pregar um papelzinho, horário, como toma, se é antes da refeição, se é depois.* (P3D)

*Olha, doutora, eu não sou de pesquisar internet, essas coisas, não. Eu tenho as informações que eu tenho, são dos meus médicos que me acompanham, né?* (P4I)

*Eu já acostumei tanto com a doutora que eu procuro vim no dia que ela tá que é pra resolver. Eu sinto assim: se eu tiver um problema, eu posso vir aqui amanhã cedo que vai ser resolvido. Eu tô muito feliz.* (P6D)

Por fim, outra forma de construir o letramento em saúde é através da percepção do próprio corpo:

*A minha cabeça fica turva às vezes e a cabeça fica ruim, aí eu sei que ela tá um pouquinho alta.* (P2D)

*Às vezes, um pouquinho de tontura., náusea, de falta de ar. Às vezes, taquicardia* [...] *até que eu fui descobrindo que era da ansiedade.* (P5I)

*Porque ela subindo descontrolada te causa ruim, causa náuseas. E ela baixa, ela me dá uma fome desesperadora, como até coisa que eu não gosto. E eu sinto muita fraqueza, às vezes dá até uma impressão que eu vou desmaiar de tontura.* (P8I)

### O diálogo entre a construção do conhecimento em saúde e as ações de cuidado dos pacientes

Quando a construção do letramento em saúde se dá principalmente através das relações interpessoais, o cuidado em saúde é atrelado a essas relações:

*Porque a minha vizinha, ela era assim. Eu não tô sentindo nada, não vou tomar. Eu também era desse jeito* [...] *mas aí, ela deitou, deu um derrame facial. Aí, daqui pra cá, ah, eu não fico sem meu remédio de jeito nenhum.* (P6D)

*Eu quero que eles tenham orgulho de mim, porque eles falam que ficam lá pensando em mim aqui.* [...] *meu filho falou: “Ó, vem aqui que a gente vai comprar os remédios pra senhora”* [...] *eu peguei e falei: “Bom, eu não vou decepcionar ele, né? Eu vou fazer certinho”.* (P6D)

[...] *ela descobriu que ela estava com câncer, né? Já era tarde, entendeu? Então, até por ter acompanhado ela com tudo isso, eu procuro cuidar bem da minha saúde, assim, o tanto que eu posso, sabe?* (P6I)

*Porque a minha vizinha, ela foi fazer, porque eu não tinha ninguém pra ir comigo, né? Então, a minha vizinha tá fazendo, porque ela tá pré-diabética e precisa fazer e ela tá me levando. Porque aí eu sozinha, eu tenho medo, eu não vou* [...]. (P2D).

*Aí, eu conversando com uma amiga minha, onde ela falou: “Não, Regina, não tem perigo, elas não deixam você afogar”. Passou confiança* [...] *ela falou: “Você pode ir comigo, a gente vem junto”* [...]. (P2D)

*A minha filha tá fazendo medicina, ela pega muito no pé da gente, ela vigia a gente de perto, né? Ela vê como tá, ela vê todos os exames, entendeu? Ela vê o que tá comendo, o que não tá comendo, ela fica de olho. Ela vigia muito a gente também.* (P6I)

*Eu escuto lá no bar, né? Que tem um monte de gente que tem diabetes lá, por isso que eu não entendo o negócio de diabetes. Eu não posso comer doce, não posso nada. Já tem outros que vai lá, que bebe pinga com coca e é diabético também. Por que um é diferente do outro?* (P6D)

Apesar das mídias sociais aparecerem como colaboradoras para a construção do conhecimento em saúde, elas não aparecem como auxiliadoras do processo de cuidado. Já a relação do conhecimento adquirido por profissionais de saúde aparece como importante colaborador, e é permeada por fatores como vínculo, escuta, enxergar o profissional como figura de autoridade e pela linguagem corporal e verbal do profissional:

*Ela pediu só os exames. Falou: “Tem que fazer tal coisa”. Eu fico realmente abatida, preocupada* [...] *causa o medo de fazer o exame e descobrir* [...] *então, eu falo assim: “Eu não vou fazer esse exame”* [...]. (P4D)

[...] *uma decisão que eu tomei em conjunto foi eu e minha psicóloga, que a gente estava discutindo os próximos passos, e eu percebi que o lugar que eu estava morando não estava bom. Fui lá e me mudei de endereço, mudei as atividades do dia que eu estava fazendo. Com o tempo, voltei a acordar mais cedo, fazer caminhada, exercício.* (P1I)

[...] *e eu tava trabalhando normalzinho de tudo, e chamaram pra eu fazer o exame periódico* [...] *o médico tirou a pressão, ouviu o meu batimento cardíaco, aí já falou pra mim que eu tinha que esperar, porque tinha dado uma alteração na pressão* [...] *atendeu uma outra pessoa lá rapidinho e já mandou chamar a ambulância, falou que eu tinha que ir embora, direto pro pronto-socorro, aí eu achei que eu tava até morrendo, paralisei* [...]. (P5D)

*Eu sinto bem, ele é especialista, ele sabe, estudou, né? Então, ele me orientou a mudar, eu tenho que mudar, né?* (P3D)

[...] *porque ele está ali se disponibilizando e vendo parte do processo que eu estou passando. Então, eu creio que está me receitando porque vai me fazer bem, né?* [...]. (P11D)

Nessa direção, a relação profissional-paciente muitas vezes dá início à motivação ao cuidado em saúde, e as sensações percebidas no corpo, assim como as relações interpessoais, funcionam como facilitadoras ou dificultadoras da manutenção desse cuidado:

*Agora, eu tô participando do grupo, né? Grupo de mulheres. É uma coisa que tá me ajudando muito a ter técnicas, né? De meditação, de respiração, a gente conversa. Então, isso tá me fazendo bem. Tô passando por consultas individuais com ela também.* (P11D)

*Ah, eu procuro, aos finais de semana, lazer com a família, dar uma atenção melhor para os filhos* [...] *porque eu percebo que a parte emocional, a ansiedade é um fator que altera bastante* [...] *pelo menos, no final de semana, eu tento fazer algumas coisas diferente* [...] *fazer caminhada, sabendo o que a gente tem que fazer para sentir melhor.* (P7D)

*É, eu estou fazendo caminhada e inclusive o meu médico psiquiatra orientou que eu fosse para uma academia, porque eu estava muito quieta. Mas do contrário, as terapias eu estou indo. Eu não posso ficar sem. A gente tem grupos, um grupo maravilhoso de apoio, então eu não deixo de ir. Se eu deixasse de ir, eu não estaria mais aqui* [...]. (P4I)

*Porque se existe um tratamento, um cuidado para isso, por que não fazer? Caminhar um pouco, comer mais fruta* [...] *e ter tranquilidade. Procurar manter a calma também, que isso contribui muito para essas sensações.* (P7I)

*Eu parei por minha conta, na hora que eu vejo que estou ficando muito ruim, eu choro, e aí passa um pouquinho* [...] *e o remédio ele melhora uma coisa, mas atrapalha a outra. A parte de namorar, eu não tinha vontade nenhuma. Então, eu acho que o remédio ajudou naquele momento que eu estava chorando demais, toda hora, mas eu acho que agora não tem necessidade* [...]. (P10I)

Ao contrário das mídias sociais, que aparecem como colaboradoras para a construção do conhecimento em saúde, mas não para o cuidado, a fé e a religião aparecem como fatores de auxílio no cuidado em saúde:

*Eu posso dizer para você que é todo dia buscar a nossa fé* [...] *porque a gente sem fé não chega a lugar nenhum* [...] *a gente lê na Bíblia, lê na palavra, buscando um lugar para ir. Acho que esse é o ponto fundamental. Não só para isso, como para todas as questões da nossa vida.* (P7I)

*Eu, graças a Deus, me livrei de tudo isso, porque eu busquei na minha fé* [...] *lógico que tem uma ajuda psicológica e psiquiátrica de doutores capacitados para isso. Mas fundamentalmente é essa busca, é um conjunto de coisas que você tem que fazer.* (P7I)

## DISCUSSÃO

Neste estudo, percebe-se o alinhamento com o manual da OMS referente às dimensões de como os usuários dos serviços de saúde adquirem conhecimento e os aplicam em práticas de autocuidado. Percebemos que a aquisição do conhecimento se dá predominantemente por meio das interações sociais de maior proximidade, incluindo amigos e familiares, sobretudo se esses últimos são profissionais de saúde ou se esse aprendizado emerge da experiência do indivíduo como cuidador de algum familiar com uma condição de saúde semelhante à sua. O diálogo permite que os indivíduos compartilhem suas experiências, anseios, busquem apoio emocional e aprendam a partir da experiência do outro, demonstrando, conforme os manuais da OMS, que o desenvolvimento do letramento em saúde é uma prática social^(2)^.

A construção do conhecimento para o letramento em saúde através das relações interpessoais motivou a maioria dos entrevistados ao autocuidado. Ao observarem complicações de doenças crônicas em pessoas próximas, passaram a cuidar melhor da alimentação ou praticar atividade física. No entanto, para alguns, essa relação gerou ceticismo em relação às informações fornecidas por profissionais de saúde, confrontando-as com a realidade vivida por seus familiares. Uma participante deste estudo mencionou um familiar com hábitos alimentares não saudáveis que teve uma longa vida, sem desenvolver diabetes, questionando a relação entre hábitos saudáveis e doenças crônicas.

Este resultado vai ao encontro dos estudos voltados a compreender o letramento em saúde de indivíduos^(1,2,14)^. O conceito de mediadores do letramento em saúde descreve pessoas que tornam o seu letramento em saúde disponível a outras, de maneira formal ou informal, e ressalta o suporte social como um dos mediadores mais importantes no letramento em saúde^(14)^, evidenciando o que o manual da OMS em letramento em saúde alerta: o fato de o letramento em saúde não ser uma tarefa individual e sobre os amigos e familiares se comunicarem com os profissionais de saúde em nome e em colaboração com os pacientes que estes atendem^(2,14)^. A proximidade das relações interpessoais também foi ao encontro daquelas encontradas neste estudo, sendo que a família nuclear é a principal mediadora da construção do letramento em saúde, seguida de amigos e, por fim, colegas de trabalho e membros de grupos de suporte^(1)^.

Esses achados destacam a importância de iniciativas como o Modelo de Cuidado Centrado no Paciente e na Família (MCCPP)^(15)^. No entanto, estudos recentes mostram a dificuldade dos profissionais de saúde em incluir pacientes e familiares nas decisões de cuidado. No Brasil, pesquisas indicam a dificuldade de integrar o MCCPP aos cuidados do paciente^(15,16)^, além de contar com um número de estudos insuficiente na área. Outro ponto importante na relação entre a construção do conhecimento em saúde e as relações interpessoais é a importância de estratégias dentro da comunidade do paciente. Pacientes que participaram de grupos de educação em saúde aprenderam mais sobre suas condições do que aqueles que não participaram^(1,14,17,18)^. As intervenções que promovem suporte em grupos são também enfatizadas pelo manual de letramento em saúde da OMS^(2)^.

Este estudo, como em outros sobre letramento em saúde, evidenciou que a tecnologia digital e as redes sociais são ferramentas fundamentais na disseminação de informação de saúde pública^(1,14,19,20)^. Alguns pacientes entrevistados consideram as mídias uma das principais fontes de aquisição de informação, devido à rapidez e acessibilidade dos conteúdos. No entanto, há uma dualidade, uma vez que a população deste estudo buscou confrontar as informações digitais com aquelas fornecidas por profissionais de saúde de confiança, percebendo-as como confusas, contraditórias ou mesmo falsas.

Sendo assim, os resultados deste estudo corroboram pesquisas anteriores, ao identificar as mídias sociais como fator importante no desenvolvimento do letramento em saúde. No entanto, neste estudo, as mídias são vistas com desconfiança e aparecem como secundárias às relações interpessoais. No entanto, como o uso das mídias sociais aparece nas falas dos participantes, é evidente a importância de utilizá-las como aliada ao cuidado em saúde. Estudos nacionais e internacionais mostram que as mídias sociais podem fornecer informações confiáveis, formar comunidades de suporte entre usuários com as mesmas condições de saúde, facilitar mudanças de comportamento necessárias para manejar condições crônicas, treinar profissionais de saúde, acompanhar e monitorar hábitos dos pacientes^(1,19,20)^.

Em consonância com estudos sobre letramento em saúde, os participantes deste estudo destacam o papel do profissional de saúde como facilitador do processo de aprendizado, sendo crucial uma boa relação médico-paciente para que o indivíduo confie nas informações recebidas. A qualidade da relação profissional saúde-paciente se relaciona à confiança, empatia, comunicação, escuta e compartilhamento de informações. Contudo, estudos mostram que nem todos os profissionais apoiam o desenvolvimento do letramento em saúde ou incentivam os pacientes a interagir com a informação antes de escolherem o tratamento. Alguns criam barreiras que impedem o desenvolvimento de competências de autocuidado^(1,14)^.

Neste estudo, pacientes destacaram que a observação da linguagem corporal e verbal dos profissionais de saúde é essencial para a tomada de decisão, gerando confiança ou descrédito. Pacientes com menor confiança e vínculo com profissionais de saúde apresentam maiores níveis de hemoglobina glicada e mais eventos cardiovasculares^(21,22)^. As principais barreiras para o letramento em saúde são a baixa habilidade de comunicação de profissionais de saúde, fazendo com que os pacientes sintam que não receberam informações o suficiente e não foram ouvidos. Alguns pacientes mencionam que suas informações não foram consideradas pelo profissional durante a consulta. Ao não levar em consideração as necessidades dos próprios pacientes, a mudança de comportamento se torna mais difícil.

Esse fato aparece nos relatos dos participantes deste estudo. Percebe-se que os participantes são mais propensos a mudar de comportamento quando percebem que as informações fornecidas pelos profissionais de saúde são construídas em conjunto, com base no vínculo e nas suas próprias necessidades. Em contraste, quando veem o profissional apenas como uma figura de autoridade, os relatos de mudança de comportamento não acompanham o discurso. Nessa direção, estratégias de comunicação, como a entrevista motivacional (EM), são importantes aliadas para promover essas mudanças. No Brasil, a EM é aplicada, em suma, no contexto do uso de substâncias psicoativas, e ainda é pouco utilizada no contexto da APS^(23)^.

Outra forma de construção do conhecimento para o letramento em saúde evidenciada neste estudo foram suas próprias percepções corporais, que foram correlacionadas à determinada condição de saúde. Cabe ao profissional de saúde estimular o paciente a desenvolver a autopercepção e orientá-lo na interpretação de tais sinais e sintomas, para que sejam diferenciados sintomas habituais de sintomas de gravidade. A OMS afirma que atividades que estimulem momentos de autopercepção favorecem a construção do letramento em saúde e promoção do autocuidado, como *Mindfulness* e Ioga, que são colocadas como ferramentas importantes na construção do autocuidado^(2,17,18)^.

Por fim, a fé, alicerçada principalmente na religião cristã, aparece como um mediador da motivação para o autocuidado em saúde, fato esse não encontrado em demais estudos qualitativos sobre o letramento em saúde. Sabe-se que as práticas e grupos religiosos têm grande influência na estrutura comunitária e, por vezes, participam da promoção de programas preventivos, como centros de reabilitação, práticas de atividade física e educação em saúde^(24)^. Nesse sentido, os locais religiosos podem ser pensados para distribuição de panfletos educativos, divulgação de informações em saúde e até mesmo palco para profissionais de saúde realizarem palestras educativas.

O estudo atual destaca a necessidade de profissionais capacitados explorar e utilizar o conhecimento prévio dos pacientes sobre suas condições crônicas de saúde, especialmente suas vivências pessoais e interpessoais. É crucial implementar ações de comunicação que alcancem comunidades e disseminem informações de saúde, de forma ampla, para além das consultas individuais. A educação permanente em técnicas de comunicação e motivação para mudança de comportamento pode ser uma estratégia eficaz. As terapias complementares, especialmente as práticas mente e corpo, são importantes aliadas, pois promovem o autoconhecimento corporal e podem ser aplicadas em grupo.

### Limitações do estudo

Este estudo identificou algumas dificuldades. Primeiro, a realização das entrevistas foi prejudicada pela limitação de espaço físico, pois uma das unidades de saúde tinha infraestrutura inadequada e uma sala pequena, sem ventilação adequada, para realização das entrevistas. Por fim, o prazo de entrega do Trabalho de Conclusão de Residência, combinado com a carga horária prática exigida pelo programa de residência, impediu a exploração de outros aspectos do letramento em saúde, como comparar a construção do letramento em saúde apontado pelos participantes com as opiniões dos profissionais de saúde que atendem essa população, a fim de investigar discrepâncias e aproximações dos discursos, assim como realizar uma análise de conteúdo antes e após intervenções de educação em saúde nos mesmos participantes. Esses apontamentos podem se configurar futuros campos de pesquisa necessários no Brasil.

### Contribuições para as áreas da enfermagem, saúde ou políticas públicas

Este estudo foi pioneiro em investigar qualitativamente a aquisição do conhecimento para o letramento em saúde em pacientes da atenção primária de saúde do Brasil. Estudos com objetivos semelhantes foram realizados fora do Brasil, analisando populações com diferentes hábitos culturais, etnia, economia, geografias e práticas sociais, sem considerar as especificidades brasileiras. Isso pode explicar porque este estudo foi o único a relatar a contribuição da religião para o letramento em saúde, destacando a necessidade de mais pesquisas brasileiras. Embora os estudos quantitativos sobre o tema sejam importantes, a pesquisa qualitativa é crucial para captar as subjetividades dos discursos, essencial para entender fenômenos sociais complexos, como o letramento em saúde.

## CONSIDERAÇÕES FINAIS

Os resultados sinalizam que a análise de como ocorre a construção do letramento em saúde no manejo de condições de doenças crônicas não transmissíveis é um processo contínuo de autoconhecimento e autogerenciamento da sua própria condição de saúde. Familiares, amigos e colegas de trabalho também são os principais agentes mediadores no desenvolvimento e na prática da alfabetização em saúde, compartilhando conhecimento, experiências, habilidades e formação de rede de apoio na tomada de decisão. Por isso, compreender a dinâmica familiar permite também entender o construto da alfabetização em saúde. A relação profissional saúde-paciente se constitui um importante pilar para que as informações recebidas pelos pacientes se transformem em motivação para o autocuidado. Nesse sentido, a APS, a partir da longitudinalidade e da abordagem centrada na pessoa (MCCPP), contribui para que a relação profissional saúde-paciente não seja uma barreira, mas sim um binômio de aliança para a construção do letramento em saúde.
